# Researches, Anatomical, Physiological, and Pathological, on the Close Cavities of the Animal Economy, Natural or Accidental

**Published:** 1844-07

**Authors:** 


### ART. VI.

*Recherches Anatomiques Physiologiques et Pathologiques sur les Cavités closes, naturelles ou accidentelles, de l'Economie Animale.* Par A. VELPEAU, &c. &c.—*Paris*, 1843.

*Researches, Anatomical, Physiological, and Pathological, on the close Cavities of the Animal Economy, natural or accidental.* By A. VELPEAU, &c.—*Paris*, 1843. 8vo, pp. 208.

THE materials of this work are derived entirely from the personal observation of its author, who has therefore professedly avoided all reference to preceding inquiries, and contented himself with regarding observations analogous to his own, but of anterior date, as so many corroborations of his statements. This method holds out many advantages where the subject of investigation is intricate; and no one is better entitled to adopt such an unfettered course than M. Velpeau, whose well-known professional erudition will at once exempt him from the charge of bringing out old things as new, through ignorance of the labours of his predecessors. We shall make our notice of his work conformable to the spirit in which it is written, and confine ourselves to an examination of the reality of the facts adduced, and the validity of the conclusions derived from them, with as little reference as possible to the history of the subject. The contents of M. Velpeau's book are rather startling, for they amount to no less than an attempt to subvert certain positions which are generally reckoned among the best established in anatomy and physiology.

The First Chapter is on the "anatomy of the close cavities," and its scope is to demonstrate the apparently singular proposition that serous and synovial membranes, as distinct tissues, have no existence, and consequently that the notion of close cavities formed by such membranes, is entirely devoid of foundation.

In support of his doctrine, M. Velpeau appeals to the development of the parts in question in the fetus, as well as to a careful investigation of them in the adult. From the examination of ten embryos, aged from fifteen to thirty days, he concludes that, at the end of the third, and often even of the fourth week of intra-uterine life, the free surfaces present no appearance of membrane. The whole body seems to consist of a homogeneous, gelatiniform, and fragile substance. The cavities present everywhere simple surfaces, and are nowhere lined with membranes consisting of a distinct tissue. There are no laminæ, or membranes capable of isolation. The whole reduces itself to *surfaces*, and *parenchymata*. We see a cutaneous surface, a mucous surface, and serous surfaces, but nothing which can justify the expressions "cutaneous membrane," "mucous membrane," or "serous membrane," whether in the head, chest, or abdomen. During the subsequent periods of fetal life, the parietes of the close cavities assume, in the course of their development, some of the characters of membranes, in certain regions of the body, or certain portions of their own extent; but these characters, which become more and more distinctly marked, even to adult age, and some of which endure even to the last term of organic evolution, are wanting completely in many of the cavities in question, and are not to be found in all parts of any one of those cavities, at whatsoever period of life they may be sought for. (pp. 2-3.)

M. Velpeau then enters minutely into the anatomy, in the adult, of the internal surfaces of the cranial, spinal, thoracic, and abdominal cavities, of those of the joints, of the tendinous sheaths, and of the bursæ mucosæ. As the peritoneal sac seems, on the whole, to afford the most unequivocal instance of a distinct membrane forming a shut cavity, we extract our author's account of it as an example of his manner of treating the whole subject.

"There is no part of the body where it is possible to detach such extensive layers of serous membrane as in the interior of the abdomen. Nevertheless, even there, the peritoneum does not exist indiscriminately at every point of the cavity called 'peritoneal.' The anterior and posterior surfaces of the stomach, the convex part of the small intestine, the cells of the large intestine, the posterior surface of the bladder, the two surfaces of the uterus, and the exterior part of the liver—none of them admit of the actual separation of a purely serous membrane. On the moveable viscera having a fleshy fibre, the serous surface being doubled, (united,) with a pretty thick fibro-cellular layer, may decidedly be detached as a membrane, but it is then clearly a cellular layer become serous on its free surface, and not a serous membrane properly so called. On the liver, an organ of which the tissue is at once dense and fragile, the serous surface evidently forms part of the subjacent layer which is itself continuous with the parenchymatous texture of the biliary organ. The same remarks apply, at all points, to the serous surface of the uterus. The ovary, the size of which does not vary continually like that of the intestines, presents, in like manner, a serous surface merely, instead of a real membrane. Behind the linea alba, in the vicinity of the umbilicus, the serous surface is so completely confounded with the other tissues of the abdominal parietes, that it is impossible to separate them otherwise than by the artificial formation of a *traumatic* peritoneum. It is evidently the uniform aspect and *unquestionable con-*

*tinuity of the whole serous surface* of the parietes of the serous cavities that has caused them to be described as so many distinct membranes. As this surface belongs to real membranes at certain points, it has thence been concluded that the membrane must exist also in parts where it could not be at all detached. To render manifest the error of such a supposition, we have only to recollect that the serous membranes do not exist prior to the organs invested by them; that the most evident of them become distinct only at a very advanced period of the embryonic state; and that all these surfaces become gradually established in the place where they are to remain, instead of applying themselves to it like an expanded veil." (pp. 9-11.)

It would be occupying our space unnecessarily, to follow M. Velpeau in his description of the serous surfaces in other cavities of the body, since his mode of demonstration and reasoning is precisely analogous to that contained in the foregoing passage; for further anatomical details, we therefore refer the reader to the work itself.

With respect to the synovial cavities, in like manner, M. Velpeau maintains that, instead of the supposed continuity of membrane throughout their inner surface, there are few vestiges of distinct membrane at any point of it. Such membrane is nowhere to be found on the free surface of the cartilages, and it is equally absent on the internal surface of most of the ligaments. In the knee, for example, if we seek for synovial membrane behind the patellar ligament, or the termination of the muscles of the thigh, we shall be unable to isolate it as a distinct membrane. "The same holds true opposite to (on the articular surface of) the internal lateral ligament and the other fibrous layers of the circumference of the joint. Outwardly, on the contour of the cartilaginous surfaces, in the grooves which separate the fibrous envelopes of the articular heads, we scarcely meet with a real synovial membrane; again, it appears to form part of the folds, and fringes, and cellulo-adipose fasciculi, called synovial glands, on several of these points." (pp. 11-2.)

This description is anything but lucid; we have therefore rendered our author's words into English as closely as possible, leaving the reader to determine their precise signification for himself. Indeed we cannot help remarking that the general style of this work falls far short of the perspicuity and neatness which usually characterize French writings on medicine, and form so agreeable a contrast to the clumsy and confused diction of too many of our own works on that science.

In the fifth section of this chapter, M. Velpeau treats of the membranes supposed to line "accidental close cavities." Such cavities he divides into "functional" and "pathological." The functional close cavities consist of, 1, *Serous cavities*; 2, *Articulations*; 3, *Cellular cavities*.

1. *Accidental serous cavities.* These are formed, according to M. Velpeau, when an ovary, a loop of intestine, a knot of epiploon, or any other viscus, passes through a fissure of the peritoneum and abdominal muscles, so as to fix itself for some months or years under the skin. When this takes place, we may be certain that a serous cavity will establish itself around the displaced organ, and the author states that he has frequently observed such an occurrence in cases of hernia, and some varieties of hydrocele. (pp. 33-4.) We should have been glad if M. Velpeau had adverted more particularly to the nature of these cases, which must, of necessity, be rare, and of which, as here alluded to, it is not easy to form any distinct conception.

These accidental serous cavities present the same characters as the normal, that is to say, they do not exist as independent sacs, but merely represent the surface of the neighbouring tissue, which has become smooth and polished from accidental causes.

2. *Accidental articular cavities.* Close cavities connected with the articulations are accidentally formed, as a consequence either of a luxation or a fracture. These we need not dwell upon, as they are perfectly familiar to surgeons; the only point to be noticed is the alleged absence of any distinct lining membrane, the inner surface of these cavities being, according to M. Velpeau, nothing more than the polished surface of the textures which enter into their composition.

3. *Accidental cellular cavities.* Accidental close cavities are nowhere so frequent, says our author, as under the skin. Wherever the skeleton forms a permanent projection at any point, if any part of the body endowed with resistance has to support long-continued or often-repeated pressure or friction, we may be sure that a mucous (synovial) cavity will there be formed. It is thus that such cavities occur on the back of porters, on the acromion of persons whose shoulders have often to sustain burdens, on the angle of the scapula in those who make use of bricoles, scuttles, &c., on the anterior part of the sternum in joiners and others, on the malleoli of tailors, on the hump of hunchbacks, on the salient points of club-feet. M. Velpeau has known such cavities to form on the body of the clavicle, on the posterior surface of the forearm, on the internal surface of the tibia, and on the crest of the ilium, in persons who have had these parts subjected to frequently repeated friction and pressure. These cavities, like normal ones of the same character, are destitute of investing membrane.

The pathological close cavities are very numerous, comprehending every species of abscess, cyst, and morbid deposit, but it is to morbid close cavities analogous to the cavities of the joints and tendons, and those which are subcutaneous, that M. Velpeau especially directs our attention. These cavities are observed in the cellular tissue, in certain glandular bodies, and in the lymphatic ganglia.

*a. In the cellular tissue.* Morbid close cavities appertaining to this tissue, have been described particularly under the name of serous cysts. There are few parts in which they have not been found, and their dimensions are extremely various. When they are situated amidst a lax and abundant cellular tissue, they may indeed be detached in the form of distinct sacs, but this simply as any layer of cellular membrane may be separated from the adjacent layers. But where these cavities are surrounded by dense tissues, they are represented, through a greater or less portion of their extent, by the substance of the tissues which they appear to line. They are, in effect, enlarged cells, or natural interstices, the parietes of which have been expended by the accumulation of the liquid with which they are filled.

*b. The glandular bodies.* The close cavities occasionally formed in the glandular bodies are adduced by M. Velpeau as illustrating his doctrine much better than those of the cellular tissue. In the thyroid body, the breast, the testicle, in which these cavities are often met with, "it is sufficient," he says, "to open one's eyes, in order to perceive that they have never existed in the capacity of membrane; that their surface makes part of the tissue of the gland itself; and that the pellicle which may occasionally be isolated from it, has been formed there in a manner similar to that which forms part of the cavity of an aneurismal sac, in which the blood has continued to circulate." (p. 37.) This fact, our author states, he has often verified in both lobes of the thyroid body, and at various points in the substance of the breast. Close cavities occurring in the ovary seem to M. Velpeau to afford unanswerable evidence of the truth of his ideas with respect to close cavities in general, for we here find real cysts, like hydatids, which are readily detached from the tissue of the organ, contrasted with close cavities, the surface of which is inseparable from it, and forms part of it. (p. 38.)

*c. Ganglial cavities.* Close cavities of this kind have been observed by M. Velpeau under the jaw, in the region of the parotid gland, in the carotid canals, in front of the larynx, in the supra-sternal fossa, in the axilla, at the bend of the arm, in the groin, in the ham, and in the interior of the pelvis. These cavities, M. Velpeau observes, present two modifications.

"1. Some of them remaining imbedded in the substance of the ganglion, resemble the close cavities of the thyroid gland; no portion of their circumference, or of their extent, can convey the idea of an ampulla (pouch) or of a membrane. They may be exactly compared to cavities hollowed out in plaster, paste, or any other inert substance. 2. The close cavity, originating near some particular point of the circumference of the ganglion, hastens to escape from the inclosure in which it was formed, to augment itself at the expense of the neighbouring layers: in this case, the ganglion, which is always to be found at some point of the cavity, prevents it from being confounded, on the one hand, with a cavity purely cellular, and, on the other, with the central cavities of parenchymatous organs." (p. 39.)

In neither of these modifications, according to M. Velpeau, can the close cavity be separated from the neighbouring tissues, of which it is, in effect, a portion.

Chapter II is on the "evolution and functions of the close cavities of the animal economy." M. Velpeau here states that the observations now brought forward form part of a general work on ovology, founded directly on observation; and of which another part, namely, that relating to the envelopes of the fetus, the placenta, the vesicles, the umbilical cord, and the external surface of the embryo, has already been published in an isolated form. If our review of the work before us were intended to be strictly analytical, this chapter would demand our special attention, inasmuch as it involves some anatomical details into which our author affirms, and we believe justly, that he has himself been the first to enter. On the whole, however, the tendency of these details being merely to illustrate those statements respecting the early condition of the internal surfaces of close cavities, which were introduced at the commencement of the present article, we are content to recommend them to our physiological readers as an original and careful investigation of the state of the surfaces in question, at various successive periods of intra-uterine life: they do not admit of condensation, and to insert them in full would swell this article to inordinate dimensions. We have the less hesitation in omitting a notice of them, because, as above stated, the inferences derived from them have already been distinctly announced, while the accuracy of the details themselves will be better tested by anatomical research than by any remarks of ours.

Chapter III is on the "diseases of the close cavities." It opens with the following proposition: "No disease originates, properly speaking, at the surface of close cavities; it is always the tissue of which these surfaces form part that is the primary seat of the malady."

"Such a proposition," says M. Velpeau, "which in the eyes of some persons might appear a paradox, if not a mere play upon words, is worthy, in my opinion, of serious discussion." (p. 96.)

Now, notwithstanding the ingenuity of our author's illustrations, we must confess that this same question of surfaces appears to us to partake largely of the nature of a logomachy, and the reason of this we apprehend to be that M. Velpeau has not sufficiently distinguished between a mathematical surface and a surface in the lax and common acceptation of the term. Viewing the thing mathematically, it is quite evident that no morbid action, and no action of any kind, can commence in a surface; for a superficies being, "that which has only length and breadth," is an idea, not a material substance, inasmuch as every portion of matter is extended in the three dimensions of length, breadth, and thickness. But when we speak, in ordinary language, of a "membranous surface," we mean the visible and tangible expanse of a thing which has thickness, however small that thickness may be.

This being premised, we would state the question as follows. It cannot be denied that the serous or synovial surface of a cavity exercises a function that would not be exercised in its absence; it must therefore have a peculiar organization adapting it to that function. Does this peculiarity of organization belong to a distinct and isolable membrane, or is it the result of a change which takes place in the structure of a subjacent tissue as it approaches its free surface? This is a point for the anatomist to decide. Without professing to have entered into any recent investigation of it with reference to M. Velpeau's doctrine, we are strongly inclined to believe that he is, to a considerable extent, correct in his anatomical views; and that the lining membranes of close cavities cannot be fairly separated from the tissues which they invest at so many points, as anatomists generally imagine. But, admitting the truth of our author's observations in their fullest extent, and taking it for granted that the so-called membranous surfaces of close cavities are not membranes at all, but that the peculiarity of organization which fits them for their function, resides in the tissue subjacent to the surface to a small portion of its depth; these things, we say, being admitted, ought we to consider the surfaces in question as distinct tissues, or as part merely of the various tissues which form the parietes of the cavity? In a word, is the recognition of serous and synovial membrane as distinct tissues to be made contingent on their separability from the parts which they invest? It seems to us that the claims of these tissues to independence is in no way affected by their mechanical connexions, nor by the fact of their imperceptible transition into other tissues. We understand, by a distinct tissue, an organized structure, which presents similar or nearly similar characters, both physical and vital, in whatsoever part of the body it may be found, or howsoever it may be distributed in relation to the structural anatomy of parts. Who can separate the tendinous from the fleshy fibre of a muscle? Yet who would maintain that tendon and fleshy fibre are not distinct tissues? The real upshot, therefore, of

M. Velpeau's observations is merely that the internal surfaces of close cavities are not generally separable in the form of continuous membranes as anatomists have imagined; but this fact though admitted, would leave their established relations to general anatomy and physiology nearly untouched.

But we proceed now to examine more particularly the pathological views of M. Velpeau.

"In purely serous cavities," he says, "in the abdomen, and especially in the chest, the tissues of which the serous surface forms the free boundary, are so vascular, and penetrated with liquids, that their inflammations seem to invade, by a similar mode of attack, the surfaces called 'peritoneum' and 'pleuræ,' and appear actually to have their starting point in the parietes (internal surface) of the cavity rather than in the layers situated beneath those parietes (that surface); but it is sufficient to reflect for a moment on the fact, that the vessels and nerves which always proceed from the deep towards the superficial parts, have their course necessarily arrested under the serous surface, whither they come to have their extremities covered; for, as every inflammation requires a vascular and nervous system for its establishment, it follows that inflammations cannot originate in the surface of serous cavities. At the same time, as the inflammatory action quickly modifies (the state of) the sanguiferous capillaries and the cellular texture of the organic layers in which it takes place, it reacts with sufficient rapidity on the serous surface to destroy almost immediately its smooth and humid aspect, and to render it impossible to recognize." (pp. 96-7.)

But we submit that M. Velpeau here falls into the same ambiguity in the use of the term "surface" which we just now pointed out, and all that he says amounts simply to this—a mathematical surface forms no part of that which it is supposed to terminate—a position which no one will dispute.

M. Velpeau seems indeed to have a certain misgiving on this head himself, for he adds,—

"If this doctrine appear to involve only a modification of language when applied to the serous cavities properly so called, it will suffice, in order to give a different idea of the matter, to put it in relation with the tendinous synovial cavities, and above all with the cavities of the joints." (p. 97.)

"On the supposition," says M. Velpeau, "that the synovial cavities are of the nature of sacs, pouches, membranes, sheaths without apertures, comparable by way of illustration to a man's night-cap, and thence concluding that every articulation contains a capsule which lines the free surface of the cartilages and the circumference of the osseous heads, and the interior of the adjacent fibro-cellular envelopes, surgeons have quietly settled it that inflammations, ulcerations, transformations, and degenerations of all kinds occur in the tissue of the synovial membranes, even on the articular cartilages. Accordingly, we find, even in the most recent works on surgery, chapters entitled 'cartilaginous synovitis,' 'ulcers, thickening, fungosities, transformation, degeneration of the synovial membrane of the cartilages.' It is, nevertheless, a fact that not one of these maladies ever commences on the free surface of a cartilage. The synovial cavity not appertaining to a proper membrane, and only existing between the osseous extremities at the expense of the cartilages which form its principal walls, could not be the seat of inflammation or of any other disease, unless the cartilages were themselves susceptible of inflammation or ulceration. Believing that I have elsewhere demonstrated that the articular cartilages are destitute of arterial and venous circulation, having convinced myself by numberless observations of every kind, that they are not the primary seat of the malady, I believe I am stating nothing but what is conformable to facts, in affirming that neither inflammation, nor ulcers, nor fungous diseases, nor transformations, nor degenerations of any kind exist as a primary malady on the free surface of the articular cartilages. That this assertion may not receive an interpretation different from that which I myself give to it, I will add that inflammations, ulcerations, &c. have been seen by myself as by others on the free surface of certain cartilages, so as apparently to confirm the opinions promulgated according to the doctrines of Bichat; but these lesions had not taken their starting point in the place where they were observed, and had already lost their original character. Thus, if an inflammation or any other alteration arise without the circumference of the cartilage, so as to cause the exhalation of a thin layer of plastic matter, this layer may deposit itself between two articular cartilages, become organized and vascular, and even confound itself with the surface of one of them. In this way we have a real membrane, more or less vascular, and free and mobile, in a greater or less degree, over a variable extent of the articular cavity. Nor is there any reason why such a layer should not sometimes attain a considerable thickness, and give the idea of fungosities, of vegetations belonging to the synovial membrane or to the cartilage. If the malady, once established in the tissues which form the contour of the articular extremities, be intense or long continued, it is possible also that it may vascularize them by degrees, so that the substance of the cartilage shall begin to undergo a real organization, and the whole shall at last become gradually organized from the circumference to the centre; but this production of vascularity, this morbid organization, by no means proves that there is a synovial membrane on the diarthrodial cartilages, or that inflammation ever originated on an isolated layer of those cartilages." (pp. 97-9.)

Here, fortunately, we get rid of the question of "surfaces," which has hitherto cast its unsubstantial film over our reasoning, and come to a point of morbid anatomy, which, however, will require further and more exact observation for its solution. But we cannot here suppress our wonder at M. Velpeau's conclusion, that cartilage is not an organized substance, because it has no visible vessels or nerves; a conclusion opposed to the whole scope of recent physiological research. A much more singular position, however, is that a plastic layer situated between two articular cartilages, and which has become organized and vascular, may confound itself with the surface of one of these cartilages, which is, by hypothesis, an inorganic substance. But even this is surpassed by the affirmation that intense or long-continued disease of the tissues surrounding the articular extremities may endow the latter (unorganized bodies according to our author) with vessels, and convert them into organized bodies. Suppose the articular extremities of the bones to be covered with some body that is really not endowed with organization—as caoutchouc for example—does M. Velpeau imagine that a lining membrane could confound its substance with the surface of the caoutchouc, or that vascular parts could penetrate it with their vessels, and communicate to it organization and vascularity, whether morbid or healthy? For our own part, we believe that articular cartilage does possess organization. We believe so, among other reasons, because it is tinged, like bone, by certain colouring matters introduced into the blood, and becomes yellow in jaundice, which would not be likely to happen with so dense a substance as cartilage if it were not organized, because it retains its shape and texture unimpaired while continually exposed to the action of liquids and to friction, which no unorganized body could do unless it were of a stony hardness, and the friction were against bodies much softer than itself; lastly, because we think we have repeatedly seen ulcerations penetrating into its substance, and granulations springing from it: we say we *think* we have seen these things, for other observers, like M. Velpeau, have derived a different impression from the exercise of their vision, which shows that there must be something in the conditions of the case that detracts from the usual certainty of ocular demonstration.

But though it were admitted that articular cartilage had no organization, it seems to us that the admission would be fatal to our author's view of the constitution of the synovial surface investing it. We find, eliminated from this surface a very peculiar secretion, which certainly differs from every other animal fluid. But, according to M. Velpeau, synovia is not secreted by the so-called synovial membrane, but by *cartilage*, of which the supposed membrane is merely the free surface. Again, articular cartilage has no organization; how then can it secrete? M. Velpeau is thus placed between the horns of a dilemma: either the synovial surface is a distinct tissue, or articular cartilage is an organized substance. We speak not here, be it remembered, of the separability or inseparability of the synovial surface as a distinct membrane, a point which appears to us to relate to structural rather than to physiological anatomy.

But it is now time to notice M. Velpeau's views with respect to the obliteration of close cavities by the artificial production of inflammation within them. After some general remarks on the effects of blood and pus effused in the close cavities, M. Velpeau proceeds as follows:

"The organic process, known under the name of adhesive inflammation, is, in some sort, peculiar to the cellular tissue. Wherever it exists alone (apart from other morbid actions), it is followed by the confusion and permanent soldering together of the neighbouring layers which have been its seat. It precedes, and generally surrounds purulent inflammations, and marches, as it were, before them, making incessant efforts to restrain and circumscribe them, and to preserve the surrounding layers from their ravages. It is, in a word, a protective process, an effort to circumscribe, within the smallest possible space, the heterogeneous matters which are developed among the tissues. But this inflammation is developed the more readily in close cavities in proportion as their parietes are more smooth and completely serous. There, as in the cellular tissue, it causes the opposite walls, which are in contact with each other, to become glued together and united, returning, in some sort, to their embryonic condition, and thus effects a complete obliteration of the cavity. Adhesive inflammation, therefore, purely adhesive inflammation, is the thing most rationally to be wished for in the instance of dropsy or sanguineous effusion in the close cavities. Nevertheless, since this inflammation is in itself productive of great danger, when it occupies very large cavities, as the peritoneum or the pleuræ, it ought not to be rashly and indiscriminately excited in all cases. We know, moreover, that adhesive, becomes easily converted into purulent inflammation, under certain influences. The problem, then, which is now to be resolved, is the following: To excite in close cavities affected with effusion an irritation which shall be *always adhesive*, and which shall *never become purulent*.

"The solution of this problem involves several data. The more the affected fluid resembles serum, the more easy it is to procure adhesive inflammation; but, on the contrary, the more it resembles pus, the less chance there is of escaping purulent inflammation. We hence perceive the propriety of endeavouring to reduce the effused fluid to the condition of serum, if its original character be not serous. In a certain number of cases, this result is obtained by emptying abscesses several times, at intervals, by a simple puncture. The same is the case with accumulations of blood. The cavity suddenly relieved from the presence of the purulent or sanguineous effusion, becomes filled anew, but exhales serum rather than a liquid analogous to that which it contained at first. This process, repeated a certain number of times under the influence of punctures, renders possible the transformation of a collection of blood or pus, into one which is purely serous, sero-sanguineous, or sero-purulent. Observations, which are already numerous, have convinced me incontestably of this fact, in most regions of the body." (pp. 106-7.)

Arguing from the effect of irritating injections in hydrocele, M. Velpeau felt inclined to try an analogous practice in other cases of effusion within close cavities; but the various substances used by surgeons for such injections appeared to him neither sufficiently mild, nor sufficiently certain in their effects, to answer his purpose. He believes, however, that he has met with the desired agent in tincture of iodine diluted with water. Iodine, he observes, seemed eligible for another reason, namely, that serous effusions in close cavities are often connected with an infarcted state of some parenchymatous organ, in which case iodine might exert its resolvent effect beneficially. He pursued his experiments with a view to ascertain, 1st, if the tincture of iodine, like wine, will cause adhesive inflammation in close cavities; 2dly, if this tincture acts beneficially in the obstructions with which dropsies are sometimes complicated; 3dly, if like wine, it occasions mortification of tissues in which it becomes infiltrated. The first question has been for some time settled in the affirmative by the experiments of M. Velpeau, and various other practitioners. On the second point our author's experience has been equally satisfactory.

"Opportunities have not been wanting to me," he says, "of appreciating the influence of injections of this kind on the obstructions which complicate hydrocele. When the dropsy had been preceded, or was still complicated with a certain degree of infarction of the testicle, the greater part of surgeons recommended the use of general and local means to overcome the infarction, and not to resort to the vinous injection till a later period. Often, again, the infarction being considered the principal malady, the injection was regarded as useless, and, in the absence of other efficient means, the testicle was sometimes sacrificed. I have proceeded quite differently with the tincture of iodine. In hydrocele I commence always by the injection; and if the infarction, though considerable, consist not in scirrhus, encephaloid structure, melanosis, or tubercle, but is in effect merely an hypertrophy, it is almost discussed. In this manner I have obtained results altogether unexpected, in the cure of tumour which had been pronounced sarcoceles by skilful practitioners. On this point I possess numerous detailed observations." (pp. 110-11.)

With reference to the third question, namely, whether the effusion of the iodine injection into the cellular tissue is followed by gangrene, M. Velpeau observed, in seven or eight cases, that, during or after the operation for hydrocele, the injection became effused between the skin and the other tissues of the scrotum, without inducing any considerable inflammation, or at worst only occasioned a very limited suppuration. To remove all ambiguity, he injected the tincture of iodine, diluted with water, into the cellular tissue of a certain number of dogs, rabbits, and guinea-pigs, and in no instance did gangrene or suppuration, or any other bad consequence follow.

M. Velpeau recommends one third of the tincture of iodine to two thirds water as the best proportions for the injection, which he represents as rather more efficacious when used cold than warm.

He then records a few cases, and alludes to many illustrative of the success of the iodine injection in the following diseases:

1. Encysted collections of serum in the tunica vaginalis.
2. Collections of serum in the tunica vaginalis, which communicate with the cavity of the abdomen, forming what is called congenital hydrocele.
3. Serous collections within a hernial sac, whether the sac be continuous with the peritoneal cavity or otherwise.
4. Encysted serous collections of the spermatic cord.
5. Serous collections in the external genital organs of women, contained in close cavities, and resembling the last mentioned.
6. Serous collections in the lymphatic ganglia of the groin and iliac fossa.
7. Collections of purely liquid blood in the interior of the pelvis in women.
8. Old collections of blood, entirely liquid, in the tunica vaginalis.

We regret much that M. Velpeau has not thought it necessary to give any detail of the cases in which he has thrown the iodine injection into hernial sacs communicating with the cavity of the peritoneum. He has, in fact, not even mentioned their number. With reference, also, to the somewhat parallel case of congenital hydrocele, though he alludes to ten cases treated with the most perfect success, he gives no details of any one of them. We confess that nothing short of the most ample and particular evidence could convince us of the safety of M. Velpeau's practice in such cases.

At p. 125, the reader will find the case of a lady, in whom a collection of blood had formed behind the uterus, ascending towards the right iliac fossa. The diagnosis formed by MM. Velpeau, Andral, and Faivre did infinite credit to their sagacity, since the operation, performed by M. Velpeau, sufficiently evinced its accuracy; nevertheless, we think the operation was rashly undertaken, as the diagnosis could not possibly be certain, though it happened to turn out correct. The cure was completed by the use of the iodine injections.

In the same manner the author treats of the diseases of the subcutaneous and accidental close cavities. Under the head of "goitre," he gives the details of four cases, three of which were collections of fluid in the thyroid body;—the seat of the effusion in the fourth case is not distinctly indicated. One of these cases—a collection of serum in the thyroid—afforded the only instances in which M. Velpeau has known constitutional disturbance to result from the use of the iodine injection. This consisted in a febrile movement of the whole system, severe headach, indigestion, and jaundice. These symptoms lasted fifteen days. The operation was eventually successful.

We pass on to the treatment of effusions in the articular and visceral cavities. M. Velpeau, fearful of dangerous consequences, long hesitated to throw the iodine injection into the joints. He tried it first in two cases of bursal tumour in the ham, the communication of which with the cavity of the knee remained doubtful. Encouraged by the absence of any evil results in these cases, he practised the operation in one of distinct hydarthrosis with perfect success, the patient retaining the complete use of the joint. The details of this case are followed by those of six others, in which the operation was successful, and one in which it had every appearance of proving so, when the patient abruptly left the hospital. We may remark, by the way, that these cases, however interesting to the practical surgeon, are anything but illustrations of the principle with which M. Velpeau set out, namely, that the iodine injection induces adhesive inflammation between the opposite surfaces of close cavities: the only cases connected with the joints which could illustrate this operation, would be those in which anchylosis was intentionally induced by the use of the injection. M. Velpeau's cases afford, nevertheless, important instances of the power of the means in question of altering the morbid action of secreting surfaces. Connected with this subject our author introduces a controversial section relative to priority in the use of the iodine injection in diseases of the joints, and claims for himself the priority, while he accords equal originality to M. Bonnet, who was not aware of his own experiments. This is quite out of place in a work constructed on the principle announced at the commencement of the treatise before us. This principle is also left out of sight in the observations on the injection of the *visceral cavities;* for our author, having made no experiments on the human subject, merely discusses the matter theoretically, and alludes to the cases recorded by Warren, L'Homme, Jobert, and Van Roos Broock, none of which gentlemen have any business here—seeing that we are in the realms of simple observation. M. Velpeau gives us, however, the details of some recent experiments on dogs, in which the injection was thrown into the peritoneal cavity. The general results are as follows:

1. When the injection was made with a third, fourth, or fifth proportion of tincture of iodine, the animals always died; but when it was made with a seventh or less, they all recovered.

2. Death was, in all instances, preceded by symptoms of violent inflammation, either of the peritoneum or intestines. There was no reason to suppose that the animals were poisoned by the absorption of the iodine.

We now draw towards the conclusion of M. Velpeau's book. In offering a general opinion of its merits, we would observe: 1. That the anatomical views it develops are probably true to a considerable extent, but that they are not borne out by sufficient exactness of detail, parts being asserted to be inseparable, without any reference to the anatomical means used to separate them. 2. That the physiological inferences appears to us to be of little importance, because, except in reference to the acceptation of terms, they leave matters very nearly where they were before. 3. That the pathological conclusions are by no means established, but should nevertheless lead to the careful revision of some points presumed, perhaps too hastily, to be ascertained. 4. That the therapeutical portion would have been much more valuable if backed by a more exact reference to cases. 5. That M. Velpeau has done injustice to his own ideas by giving them to the world in language which is too diffuse, and yet hard to understand from its abounding with words of vague signification; lastly, that, with all its faults, the book is well worthy of perusal —a fact for which the very name of the author is a sufficient guarantee.

